# Malignant renal tumors in children

**DOI:** 10.15586/jkcvhl.2015.29

**Published:** 2015-05-10

**Authors:** Justin Scott Lee, Thomas Ray Sanchez, Sandra Wootton-Gorges

**Affiliations:** 1 Department of Radiology; 2 Division of Pediatric Radiology, University of California, Davis Medical Center and Children’s Hospital, Sacramento, CA, USA.

## Abstract

Renal malignancies are common in children. While the majority of malignant renal masses are secondary to Wilms tumor, it can be challenging to distinguish from more aggressive renal masses. For suspicious renal lesions, it is crucial to ensure prompt diagnosis in order to select the appropriate surgical procedure and treatment. This review article will discuss the common differential diagnosis that can be encountered when evaluating a suspicious renal mass in the pediatric population. This includes clear cell sarcoma of the kidney, malignant rhabdoid tumor, renal medullary carcinoma and lymphoma.

## Introduction

Renal malignancies are common in children. Favorable histology Wilms tumor (FHWT) is the most common subtype of Wilms and, fortunately, also has the best prognosis with overall survival greater than 90%. Despite advances in treatment achieved with Wilms tumor, 30% of pediatric renal tumors still have overall survival less than 70%. This emphasizes the need for development of multimodal biological treatment regimens and further understanding of prognostic biomarkers (1). Improved detection methods for different pediatric renal malignancies and subsequent specific clinical trials would be beneficial. It is paramount for clinicians to correctly identify the lesion and select the optimal treatment.

## Wilms Tumor

An impressive 87% of pediatric renal masses are Wilms tumor (nephroblastoma), which makes up 7% of childhood malignancies and is the fourth most common childhood cancer overall (2–4). Each year in the United States about 500 children are diagnosed with Wilms tumor with 80% of patients presenting before 5 years of age (5). It arises from persistent metanephric blastema, and is subdivided into FHWT, anaplastic Wilms tumor (AHWT), and blastemal-type Wilms. Children with Beckwith-Wiedemann syndrome, WAGR syndrome and Denys-Drash syndrome, have increased risk of Wilms tumor and may be screened with ultrasound up until about age 6 years.

**Figure 1.**
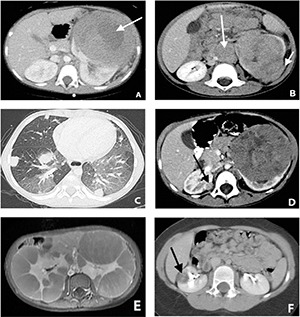
**A,** Large left renal mass in a 4-year old female with central areas of necrosis (arrow). Diagnosis was Wilms tumor on surgical pathology; **B,** Three-year old female with a left renal mass (Wilms tumor) with associated retroperitoneal lymphadenopathy (long arrow). There is also extracapsular extension of the tumor (short arrow). **C,** CT of the chest showed multiple metastatic pulmonary nodules; **D,** Five-year old female with bilateral Wilms tumor. Large left renal mass and a smaller right renal mass (arrow). Bilateral partial nephrectomies were performed; **E,** Nephroblastomatosis. Eighteen-month old female with an enlarged abdomen. Axial post contrast MRI shows bilateral nephromegaly containing multiple lobulated parenchymal masses with streaky enhancement; **F,** Six-year old female with a right renal mass (arrow). This was a renal cell carcinoma on surgical pathology after nephrectomy.

While it can be discovered during coincidental trauma in up to 10% of cases (6), Wilms tumor commonly presents as a palpable abdominal mass (palpated by parents or on physical exam by physicians); it can be associated with hypertension (in up to 25% of cases, caused by renin produced by tumor cells), and hematuria (7). The mass may contain hemorrhage, calcification, fat, or necrosis and may invade the renal vein or inferior vena cava (IVC). On imaging, the classic appearance is a heterogeneous solid renal mass with a “claw sign” indicating its origin from the renal parenchyma **(Figure 1A**. Tumor extension into the renal vein and IVC is seen in 5–10%. Wilms tumor most frequently metastasizes to the lungs **(Figure 1B,C)** (8). Wilms Tumor is bilateral in 4–13% of cases **(Figure 1D)**. A cystic variant of Wilms tumor may mimic benign multilocular cystic nephroma.

The National Wilms Tumor Study (NWTS) and Societe Internationale D’Oncologie Pediatrique (SIOP) have made large contributions to the modern multimodal treatment, which consists of surgical excision, radiotherapy, and chemotherapy (adjuvant and/or neoadjuvant). Minimally invasive laparoscopic surgery has also increased in popularity. These oncologic treatments achieve a remarkable long-term overall survival rate of 90%, however, 25% of survivors have serious chronic therapy-related health conditions up to 25 years later (9). Anaplastic, bilateral, or relapsed disease has a lower survival rate (10).

## Nephroblastomatosis

Nephroblastomatosis is a rare pre-neoplastic precursor to Wilms tumor. It is defined as multiple nephrogenic rests or abnormal foci of persistent metanephric blastema beyond 36 weeks of gestation. Normally nephrogenesis completes at 36 weeks gestation with metanephric blastema forming nephrons and eventually the renal cortex. Nephrogenic rests are present in 1% of infants at autopsy, and malignant transformation occurs in less than 1% of nephrogenic rests. They can be perilobar (at the periphery of the renal lobe) or intralobar (within the renal lobe). More than 30% of Wilms tumors arise from nephrogenic rests, which are found in nearly all patients with bilateral Wilms.

Screening is recommended at 3-month intervals for nephroblastomatosis to detect malignant transformation. Ultrasound may reduce both cost and use of anesthesia for sedation and can detect masses, however MRI is the method of choice for follow-up (11). On imaging, nephroblastomatosis appear as discrete, homogeneous, non-enhancing renal masses **(Figure 1E)**. Any rapid growth, inhomogeneity, or heterogeneous enhancement is considered worrisome for development of Wilms.

## Renal Cell Carcinoma

Renal cell carcinoma (RCC) makes up 1% of all pediatric renal malignancies. In the pediatric setting it is typically seen in Von Hippel-Lindau disease, which is associated with retinal and central nervous system hemangioblastomas, pheochromocytomas, and pancreatic neuroendocrine tumors. On imaging RCC is indistinguishable from Wilms tumor **(Figure 1F)**, though it tends to be smaller at presentation and is calcified in 25% (12). The 5-year survival rate is less than 70% (13).

## Renal Medullary Carcinoma

**Figure 2.**
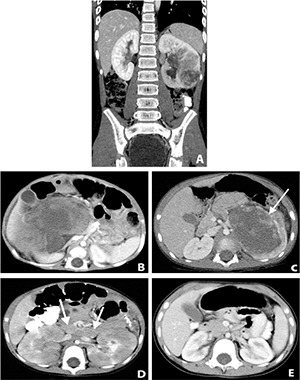
**A,** Ten-year old African-American male with sickle-cell trait. Coronal CT scan shows a centrally located and infiltrating left renal mass. Nephrectomy was performed and surgical pathology came back as renal medullary carcinoma. **B,** Large right renal mass in a 7-month old male. Nephrectomy was performed and the surgical pathology revealed a rhabdoid tumor. **C,** Two -year old male with a large left renal mass indistinguishable from a Wilms tumor. The mass was a clear cell sarcoma on surgical pathology. **D,** Four-year old female with history of abdominal enlargement. The kidneys are enlarged and show multiple hypodensities. There are also enlarged retroperitoneal lymph nodes (arrow); **E,** Follow-up CT 5 months later after chemotherapy showing normal kidneys and resolution of the retoperitoneal lymphadenopathy.

Renal medullary carcinoma is an aggressive tumor almost exclusively seen in young (age11-39years), African-American patients with sickle cell hemoglobinopathy (14). It usually presents at an advanced stage and typically poorly responds to treatment. It is an epithelial tumor arising from the collecting duct epithelium. While there is no specific imaging appearance, it is located deep within the renal pelvis and sinus **(Figure 2A)** in contradistinction to Wilms and RCC which are usually located in the renal cortex. Immunohistochemically, renal medullary carcinoma stains positive for CAM 5.5 and epithelial membrane antigen, negative for cytokeratin 34EE12, and variable for cytokeratins 7, 20, and carcinoembrionic antigen (15). Surgery, chemotherapy, and radiation have been used, however the average reported survival continues to be very poor at 15 weeks from diagnosis (16). Awareness and early detection may help increase survival and thus cytologic evaluation of urine and computed tomography (CT) imaging may be appropriate for patients with sickle cell trait presenting with persistent renal symptoms of hematuria, flank pain, and weight loss.

## Malignant Rhabdoid Tumor (MRT)

MRT is a rare, aggressive malignancy that most commonly originates in the kidney **(Figure 2B)** but can also occur in the central nervous system and other extrarenal sites. It is commonly diagnosed between 0–3 years of age. It is also associated with extensive metastasis at the time of diagnosis and can have a synchronous brain tumor. A key to the diagnosis is negativity of immunohistochemistry for SWI/SNF-related, matrix-associated, actin-dependent regulator of chromatin, sub-family B, member 1 INI1 (SMARCB1) (17). There is no standard treatment for MRT and prognosis is very poor with published overall survivals of 15–36%, however more recent treatment regimens including surgery, radiotherapy, high dose chemotherapy and autologous stem cell rescue (HDCT/ASCR) may improve survival with one center reporting overall survival of 66.7% with a median follow up of almost 2 years (18).

## Clear cell sarcoma of the kidney (CCSK)

CCSK is the second most common renal tumor in children with a mean age of diagnosis overlapping with Wilms tumor. Clinical presentation includes abdominal pain, hypertension, and hematuria. CCSK is aggressive with a unique propensity to metastasize to bone and brain, as well as lung and abdomen (19). It is virtually indistinguishable from Wilms tumor on imaging **(Figure 2C)**. Treatment consists of radical nephrectomy, chemotherapy, and radiotherapy. While prognosis traditionally has been poor, newer treatment regimens show relapse-free and cancer-specific survival rate of 100% for revised stage 1 CCSK (20) and overall survival rate for patients with CCSK of 83% (21).

## Lymphoma

Primary lymphoma of the kidney is extremely rare since the kidney has no lymphatic system. Disease involvement is mainly due to hematogenous or direct spread from adjacent lymph nodes. The kidneys are involved in 12% of children with non-Hodgkin’s lymphoma. On imaging, lymphoma may have a variety of appearances that include multiple bilateral low-attenuation masses **(Figure 2D, E)**, areas of geographic wedge shaped low attenuation, unilateral solitary low-attenuation mass, unilateral large conglomerate masses, or unilateral nephromegaly seen on CT (22). Lymphoma may mimic nephroblastomatosis, however, lymphoma occurs typically in older children and is associated with lymphadenopathy elsewhere in the body.

Treatment is mainly with chemotherapy; however careful nephrological monitoring is necessary during treatment since excretion of tumor metabolites may result in renal obstructive or uric acid nephropathy (23).

## Conclusion

Although Wilms tumor is the most common pediatric renal malignancy, it is often indistinguishable from other rare but more aggressive masses like RCC and MRT. Imaging and histologic characterization are crucial to avoid delay in management. New treatment regimens continue to improve survival rates, however further research and advancement in knowledge is still necessary to improve outcomes and decrease treatment-related long-term complications.

## References

[1] Dome JS (2013). COG Renal Tumors Committee 2013, Children’s Oncology Group’s 2013 blueprint for research: Renal tumors.. Pediatr Blood Cancer.

[2] Julian JC, Merguerian PA, Shortliffe LM. (1995). Pediatric genitourinary tumors.. Curr Opin Oncol.

[3] Ritchey ML, Azizkhan RG, Beckwith JB, Hrabovsky EE, Haase GM. (1995). Neonatal wilms tumor.. J Pediatr Surg.

[4] Grovas A, Fremgen A, Rauck A, Ruymann FB, Hutchinson CL, Winchester DP, Menck HR. (1997). The National Cancer Data Base report on patterns of childhood cancers in the United States.. Cancer.

[5] Charles AK, Vujanić GM, Berry PJ. (1998). Renal tumours of childhood.. Histopathology.

[6] Lonergan GJ, Martínez-León M, Agrons GA, Montemarano H, Suarez ES. (1998). Nephrogenic rests, nephroblastomatosis, and associated lesions of the kidney.. Radiographics.

[7] Sanchez T, Ducore J, Balagtas J, Molloy C, Wootton-Gorges S. (2014). 2014 WARM N COLD: malignant and benign renal tumors in children.. Emerg Radiol.

[8] Son J Lee EY Restrepo R Eisenberg RL. Focal renal lesions in pediatric patients. AJR Am J Roentgenol 2012 199 6 W668-82 Doi: http://dx.doi.org/10.2214/AJR.11.8082 23169739 10.2214/AJR.11.8082

[9] Dome JS (2013). Children’s Oncology Group’s 2013 blueprint for research: Renal tumors.. Pediatr Blood Cancer.

[10] Bhatnagar S. (2009 Jan). Management of Wilms’ tumor: NWTS vs SIOP.. J Indian Assoc Pediatr Surg.

[11] Sethi AT, Narla LD, Fitch SJ, Frable WJ. (2010). Best cases from the AFIP: Wilms tumor in the setting of bilateral nephroblastomatosis.. Radiographics.

[12] Zagoria R, Wolfman N, Karstaedt N, Hinn G, Dyer R, Chen Y. 1990 CT features of renal cell carcinoma with emphasis on relation to tumor size.. Invest Radiol.

[13] Leuschner I, Harms D, Schmidt D. (1991). Renal cell carcinoma in children: histology, immunohistochemistry, and follow-up of 10 cases.. Med Pediatr Oncol.

[14] Baig MA, Lin YS, Rasheed J, Mittman N. (2006). Renal medullary carcinoma.. J Natl Med Assoc.

[15] Swartz MA, Karth J, Schneider DT, Rodriguez R, Beckwith JB, Perlman EJ. (2002). Renal medullary carcinoma: Clinical, pathological, immunohistochemical and genetic analysis with pathogenic implications.. Urology.

[16] Davis CJ, Mostofi FK, Sesterhenn IA. (1995). Renal medullary carcinoma. The seventh sickle cell nephropathy.. Am J Surg Pathol.

[17] Kato M, Koh K, Oshima K, Oguma E, Uchida H, Kishimoto H, Kikuchi A, Hanada R. (2013). Long-term survivor of relapsed stage IV malignant rhabdoid tumor of the kidney.. Pediatr Int.

[18] Hong CR, Kang HJ, Ju HY, Lee JW, Kim H, Park SH, Kim IH, Park KD, Shin HY. (2015 Jan 2). Extra-cranial Malignant Rhabdoid Tumor in Children: A Single Institute Experience.. Cancer Res Treat.

[19] Malkan AD, Loh A, Bahrami A, Navid F, Coleman J, Green DM, Davidoff AM, Sandoval JA. (2015). Pediatrics.

[20] Kalapurakal JA, Perlman EJ, Seibel NL, Ritchey M, Dome JS, Grundy PE. (2013). Outcomes of patients with revised stage I clear cell sarcoma of kidney treated in National Wilms Tumor Studies 1–5.. Int J Radiat Oncol Biol Phys.

[21] Seibel NL (2004). Effect of duration of treatment on treatment outcome for patients with clear-cell sarcoma of the kidney: a report from the National Wilms’ Tumor Study Group.. J Clin Oncol.

[22] Hilmes MA, Dillman JR, Mody RJ, Strouse PJ. (2008). Pediatric renal leukemia: spectrum of CT imaging findings.. Pediatr Radiol.

[23] McHugh K. (2007). Renal and adrenal tumours in children.. Cancer Imaging.

